# Is the development of obesogenic food environments a self-reinforcing process? Evidence from soft drink consumption

**DOI:** 10.1186/s12992-021-00735-y

**Published:** 2021-08-18

**Authors:** Fabrizio Ferretti, Michele Mariani, Elena Sarti

**Affiliations:** 1 School of Social Sciences, University of Modena and Reggio Emilia, Reggio Emilia, Italy; 2 Department of Communication and Economics, Viale Allegri 9, 42121 Reggio Emilia, Italy

**Keywords:** Cross-country longitudinal dataset, Obesity, Obesogenic environments, Soft drinks, Simultaneous equation model

## Abstract

**Background:**

Understanding how the development of obesogenic food environments and the consumption of ultra-processed foods and beverages influence each other can help policymakers to identify effective ways to curb the current obesity epidemic. This paper was designed to investigate whether, and to what extent, the consumption of soft drinks and the prevalence of obesity are linked through feedback effects.

**Methods:**

An ecological study design and a simultaneous equation model were used to investigate the existence of a vicious cycle between the consumption of soft drinks and the prevalence of obesity. The analysis was based on a longitudinal dataset covering per capita sales of soft drinks, the age-standardised prevalence rate of obesity and several demographic and socio-economic control variables in a sample of 98 countries worldwide for the period 2005–2019.

**Results:**

Using a Two-Stage Least Squares (2SLS) regression model with fixed effects, we documented a self-reinforcing process that links consumption and obesity. Changes in the spread of obesity were associated with changes in soft drink consumption: a one-unit increase in the age-adjusted prevalence rate of obesity increased consumption by about 2.39 l per person per year. Similarly, as the consumption of soft drinks rose, so did the prevalence of obesity: the age-adjusted rate of obesity increased by 0.07% for every additional litre consumed per capita. Computing the impact multipliers, we found that the outcome of a one-unit decrease in the average price of soft drinks was twofold: a) the prevalence of obesity increased by around 0.17%; and b) consumption increased by around 2.40 l per person, the sum of the increase directly caused by the price reduction (2 l) and the increase due to the interplay between consumption and obesity (0.4 l).

**Conclusions:**

This study has identified a feedback loop between unhealthy habits (i.e. the consumption of soft drinks) and health outcomes (i.e. the prevalence of obesity). This interplay amplifies the impact of any exogenous changes in the determinants of consumption and obesity. These feedback effects should be considered and exploited in planning effective strategies to tackle the burden of obesity and the global epidemic of non-​communicable diseases.

**Supplementary Information:**

The online version contains supplementary material available at 10.1186/s12992-021-00735-y.

## Background

According to the World Health Organization (WHO), obesity is a major preventable risk factor for several chronic non-communicable diseases (NCDs), such as cardiovascular diseases (mainly heart disease and stroke), diabetes, musculoskeletal disorders (especially osteoarthritis) and some type of cancers [1]. In addition, recent evidence also suggests that obesity is an important parameter for Coronavirus Disease 2019 (COVID-19) risk assessment because obesity and obesity-related morbidities tend to increase the risk of severe COVID-19 outcomes including admission to Intensive Care Units (ICUs) and are associated with higher fatality rates [2].

A wide range of interrelated physical, cultural, and socio-economic factors can promote unhealthy weight gain as a result of excessive energy intake and/or insufficient physical activity [3]. People’s exposure to these factors has increased dramatically over recent decades in advanced and emerging economies [4, 5]. Because of the spread of the so-called obesogenic environments [4, 6], the worldwide prevalence of obesity has nearly tripled since the middle of the 1970s. Obesity has now become an impending global health challenge [7].

A large and growing body of literature has investigated the impact of single nutrients and different dietary patterns on unhealthy increases in weight status, focusing mainly on the role of ultra-processed foods and beverages [8–10]. These are usually calorie-dense products—high in salt, refined carbohydrates, free sugars, unhealthy fats, and additives—and their widespread availability is a prominent feature of the current obesogenic food environments [11, 12].

In this literature, the direction of causality runs from consumption to obesity; that is, variations in consumption of ultra-processed foods and beverages (the independent variable) are used to explain changes in weight status (the dependent variable) by controlling for the effects of several possible confounding factors. Conversely, the existence of a two-way relationship between the consumption of ultra-processed products and the prevalence of obesity remains largely unexamined.

**Fig. 1:**
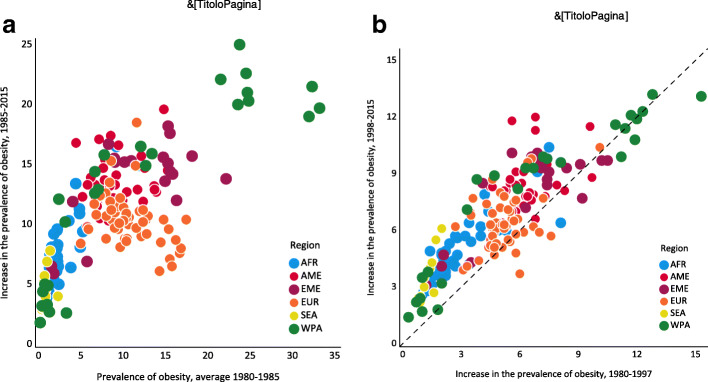
**A**. Prevalence of obesity (average levels 1980–85 and increases 1985–2015). **B**. Increase in the prevalence of obesity (1980–1997 and 1998–2015). Legends: Prevalence of obesity (Age-stand. Rate, both sexes, 18+ years, %)

However, there are reasons to suspect that the causality between the development of an obesogenic environment and the prevalence of obesity may run in both directions, meaning that the spread of obesogenic factors and the spread of obesity tend to reinforce one other. The sharp increase in the worldwide prevalence of obesity, for instance, seems to be driven by a self-sustaining process, as shown in Fig. 1A and B. These figures display data concerning the age-standardised prevalence rate of obesity (in people aged 18 years and over) in 190 countries, grouped according to the WHO’s six world regions. During the period 1985–2015 (Fig. 1A), increases in the prevalence of obesity (measured on the y-axis) were positively related to their starting levels (measured on the x-axis, by the 1980–1985 average). Except for a few European countries (some of the orange dots), nations with the highest prevalence rate in the period 1980–1985 have experienced the greatest increases in the spread of obesity from 1985 to 2015. Moreover, in Fig. 1B, changes in the prevalence of obesity during the period 1980–1997 are plotted against their changes over the next 18 years (1998–2015). The data points lie substantially above the 45-degree dashed line for almost all countries, indicating that increases in the prevalence of obesity were associated with further and even greater increases.

A possible explanation for this evidence is the existence of a vicious cycle [13]. An adverse feedback loop, wherein the adoption of unhealthy habits increases the prevalence of obesity, which in turn promotes the spread of obesogenic factors, which leads to further increases in unhealthy habits, and so on. This hypothesis suggests the following research question: Is the development of an obesogenic food environment a self-reinforcing process? In this paper, we attempt to address this issue by focusing on the role of soft drinks.

The term ‘soft drinks’ generally refers to a wide range of non-alcoholic carbonated and non-carbonated water-based (or, in some cases, milk-based) flavoured drinks, usually added with sugar or other natural and artificial sweeteners [14]. Soft drinks are ubiquitous in modern food environments [15]. Their regular consumption has been associated with an increased risk of adverse health outcomes, including overweight and obesity, type 2 diabetes, and heart diseases [16]. This is because the vast majority of soft drinks are high in free sugars and various additives, but also because they usually serve as complement-in-consumption of ultra-processed unhealthy foods and snacks [17–19].

The purpose of this study is to investigate the existence of a vicious cycle between the consumption of (and thus the demand for) soft drinks and the prevalence of obesity. To this end, we use an ecological study design and a simultaneous equation model. The analysis is based on a longitudinal dataset covering per capita consumption of soft drinks, the age-standardised prevalence rate of obesity, as well as other demographic and socio-economic control variables in a sample of 98 countries worldwide for the period 2005–2019.

We build on previous research based on similar cross-country datasets. These studies have examined the association between the consumption of soft drinks (their prices and affordability) and the prevalence of obesity [20–22]. More recently, research has begun to examine the impact of obesity in determining the market demand for soft drinks [23]. However, what remains unclear is whether, and to what extent, the consumption of soft drinks and the prevalence of obesity influence each other through feedback effects that trigger a self-reinforcing process. Unlike these previous studies, in this paper, we model the relationship between soft drinks and obesity using a simultaneous equation approach. Understanding and measuring the role of potential feedback effects may be helpful in the design of a better comprehensive strategy to curb soft drink consumption and tackle the current obesity epidemic [24].

## Methods and data

This study set out to test the hypothesis that the consumption of soft drinks and the prevalence of obesity are jointly determined. A simultaneous equation model can capture this idea as follows:

1 $$ QSD={a}_0+{a}_1 OBE+{a}_2{X}_1+{a}_3{X}_2+\dots +{a}_n{X}_n. $$

2 $$ OBE={b}_0+{b}_1 QSD+{b}_2{X}_2+{b}_3{X}_3+\cdots +{b}_n{X}_n. $$

where *QSD* and *OBE* denote the per capita consumption of soft drinks and the prevalence of obesity, respectively. In these two structural equations, the variables *QSD* and *OBE* are simultaneously determined (i.e. they are both endogenous), whereas the *Xs* represent demographic and socio-economic exogenous control variables that may affect either or both *QSD* and *OBE*. In other words, a change in soft drink consumption (Eq. 2) will cause a change in the prevalence of obesity, which will, in turn, cause soft drink consumption to change again (Eq. 1), and so forth. Likewise, a change in the prevalence of obesity, in Eq. 1), will lead to a change in soft drink consumption that, through Eq. 2), will trigger a feedback loop that promotes further changes in both the prevalence of obesity and the consumption of soft drinks. Conversely, a change in an exogenous variable (for example, *X*_1_) will not loop back through the system and cause *X*_1_ to change again.

### Data sources

In order to develop the empirical analysis, we collected secondary data from the following five sources: Euromonitor International, the World Health Organisation (WHO), the World Bank Group (WBG), the United Nations Food and Agriculture Organisation (FAO), and the KOF Swiss Economic Institute.

Passport, the Global Market Information Database of Euromonitor International [25], contains information about sales, in both volume and value, of the different types of soft drinks for a large number of countries worldwide. From this dataset, we computed the soft drink consumption per capita (*QSD*) and the average price of soft drinks (*PSD*) in 98 countries from 2005 to 2019.

Specifically, *QSD* was obtained by dividing the total sales in volume of both non-carbonated (i.e. fruit juices, ready-to-drink tea, and coffee, as well as sports/energy and Asian drinks) and carbonated (i.e. regular and diet sodas) soft drinks by the total country population. *QSD* is measured in litres per person per year and includes on-trade and off-trade sales of both domestically manufactured and imported beverages (but it does not include bottled (carbonated and still) water).

Similarly, we computed the average price of soft drinks (*PSD*) as the ratio of total sales in value to total sales in volume. Prices measured in local currencies were thus converted to international dollars—using purchasing power parity (PPP) conversion factors, provided by the International Comparison Program of the World Bank [26]—and expressed in 2017 constant prices to allow comparability across countries and over time. *PSD* is the average retail selling price per litre, including sales and excise taxes.

The Global Health Observatory [27] of the World Health Organization (WHO) collects comparable estimates of the prevalence of overweight and obesity for almost all countries worldwide. From this data repository, we retrieved the age-standardised adjusted estimates of the prevalence of overweight and obesity (*OWE* and *OBE*) among the adult population. The variables *OWE* and *OBE* are measured by the percentage of adults (aged 18 years or older) who have a body mass index (BMI) equal to or greater than 25 and 30 kg/m^2^, respectively (where BMI is defined as weight in kilograms divided by the square of height in metres).

From the World Bank Open Data repository [28], data were gathered for the following three variables: income per capita (*GNI*), the share of the population aged 65 and above (*AGE*), and the ratio of urban to total population (*URB*). It has previously been observed that soft drink affordability—defined as the ratio between the average price of soft drinks and the income of potential consumers—is one of the major factors affecting purchasing behaviour [22, 29]. Besides the market price, data on Gross National Income per capita (GNI, measured in constant 2017 international dollars) were thus included in the analysis to account for overtime within-country differences in average income (and, ultimately, in standards of living).

The share of elderly people, measured by the number of people aged 65 and above as a percentage of the total population (*AGE*), was included as a demographic driver of both soft drink consumption and population health outcomes. On the one hand, soft drinks are popular beverages, especially among children, adolescents, and young adults, who usually represent the populations targeted by the soft drink industry with aggressive marketing strategies [30, 31]. On the other hand, although the prevalence of obesity among children and adolescents has risen dramatically over the last decades, the number of elderly individuals with obesity has also increased, and it is expected to increase further as the population ages, mainly in emerging economies [32]. Therefore, increases in the share of elderly people should decrease the consumption of soft drinks and increase the prevalence of obesity.

As highlighted by prior research [33], economic and social structural changes, such as the increasing number of people who live in urban areas, typically lead to more sedentary lifestyles and consequently to a decreasing home and occupational energy expenditure. Thus, the share of urban to total population (*URB*) was used here as a proxy variable to capture in Eq. 2) these potential drivers of the obesity pandemic.

Starting from national balance food sheets, the Food and Agriculture Organization (FAO) of the United Nations [34] computes the dietary energy supply (*DES*). These are estimates of the average number of calories available for human consumption in each country (measured in kcal per person per day). An increased food energy supply has been associated with the spread of unhealthy increases in population body weight [8]. Therefore, we used these internationally comparable *DES* estimates to capture the impact of changes in food availability on the prevalence of obesity.

Finally, the KOF Swiss Economic Institute [35] has developed an index to measure the overall degree of globalisation, and sub-indices to measure the degree of the different (i.e. economic, political, etc.) dimensions of the globalisation process. In the soft drink industry, large multinational companies and specialised local firms compete in a global business environment [36]. International trade and foreign direct investment tend to increase the supply and variety of soft drinks available to consumers, whereas global advertising strategies may affect people’s health-related lifestyles [37]. We considered the role of these processes in shaping modern obesogenic environments by including the KOF Index of economic globalisation among the exogenous control variables as a potential determinant of both the consumption of soft drinks and the spread of obesity.

Overall, we collected data about nine variables—the consumption of soft drinks per capita (*QSD*), the age-standardised prevalence rate of overweight and obesity (*OWE* and *OBE*), the average price of soft drinks (*PSD*), the gross national income per capita (*GNI*), the dietary energy supply (*DES*), the share of urban population (*URB*), the share of the elderly population (*AGE*), and the degree of economic globalisation (*GLO*)—from 98 countries, where each country is observed in *t* = 15 time periods (each year from 2005 to 2019), for a total of 1470 observations. Hong Kong and Taiwan, however, were omitted from the regression analysis due to incomplete information on several variables. The resultant dataset is a balanced panel data containing 1440 observations.

A short description of each variable, along with basic descriptive statistics, is shown in Table 1. A list of the countries included in the analysis is shown in Table 1A in the Supporting Information File S1. For the entire dataset, see Table 2A in the Supporting Information File S1. The dataset is also publicly available at Mendeley Data (10.17632/hkm25rbpsc.2).

**Table 1:** Summary of variables and descriptive statistics (all countries, 2005–2019)

Variable	Description	Mean	Std. dev.	Min	Max	N. of obs.
*QSD*	Soft drink consumption per capita (litres/person/year)	75.21	49.22	1.69	270.95	1470
*OBE*	Prevalence of obesity (BMI ≥ 30 kg/m^2^. Age-stand. Rate, both sexes, 18+ years, %)	18.44	8.65	0.90	38.50	1455
*OWE*	Prevalence of overweight (BMI ≥ 25 kg/m^2^. Age-stand. Rate, both sexes, 18+ years, %)	49.20	15.35	12.2	73.5	1440
*PSD*	Soft drink price (average per litre PPP, constant 2017 international $)	3.44	1.23	1.58	15.12	1470
*GNI*	Gross national income per capita (PPP, constant 2017 international $)	23,993.90	19,745.52	892.83	97,094.19	1455
*DES*	Dietary energy supply (kcal/person/day)	2984.00	421.89	1729	3847	1470
*URB*	Urban population (as % of total population)	65.01	20.06	15.70	100.00	1455
*AGE*	Population aged 65 and above (as % of total population)	10.25	6.14	0.69	28.00	1455
*GLO*	KOF Index of economic globalisation (min = 0, max = 100)	63.08	15.79	25.5	95.3	1455

**Notes:** BMI: Body mass index; PPP: Purchasing power parity; KOF: Swiss Economic Institute

### Regression model

Using the above-described set of variables, we can rewrite the system of simultaneous Eqs. 1) and 2) as follows:

3 $$ {QSD}_{it}={\upalpha}_0+{\upalpha}_1{OBE}_{it}+{\upalpha}_2{PSD}_{it}+{\upalpha}_3{GNI}_{it}+{\upalpha}_4{AGE}_{it}+{\upalpha}_5{GLO}_{it}+{\upalpha}_i+{u}_{it}. $$

4 $$ {OBE}_{it}={\upbeta}_0+{\upbeta}_1{QSD}_{it}+{\upbeta}_2{DES}_{it}+{\upbeta}_3{URB}_{it}+{\upbeta}_4{AGE}_{it}+{\upbeta}_5{GLO}_{it}+{\upbeta}_i+{u}_{it}. $$

where the subscripts *i* and *t* refer to the country and year, respectively. The consumption of soft drinks and the prevalence of obesity tend to vary greatly across and within countries, even among countries with similar economic characteristics. Because several cultural and social unobserved factors may affect both *QSD* and *OBE*, a fixed-effects regression model was used to control for potential country-specific omitted variables. Thus, α_*i*_ and β_*i*_ in Eqs. 3) and 4) denote the time-invariant country-specific constant (i.e. the country- specific fixed effects representing unobserved heterogeneity).

The structural Eqs. 3) and 4) describe a system in which *QSD* and *OBE* are jointly determined (i.e. soft drink consumption and the prevalence of obesity are both endogenous variables). This system thus violates the classical assumption of independence between the error term and the explanatory variables. Applying ordinary least squares (OLS) directly to the structural equations of such a simultaneous system produces biased estimates because OLS regression is likely to attribute to the explanatory variable variations in the dependent variable that are actually being caused by variations in the error term. We adopted a Two-Stage Least Squares (2SLS) estimation technique to mitigate the effects of this simultaneity bias [38].

In the first stage, the 2SLS technique uses a linear combination of all the exogenous variables in the system to produce predicted values of the endogenous variables. In the second stage, these predicted values are utilised to replace the endogenous variables, where they appear on the right-hand side of each structural equation. In the first stage, the exogenous variables are thus used as instrumental variables. Valid instrumental variables must be relevant (i.e. a good proxy of the endogenous variables) and exogenous (i.e. uncorrelated with the error term). The Underidentification test is used to check the relevance of the instruments. It determines whether the variation in the instruments is related to the variation in the endogenous variables. The Sargan–Hansen J-statistic refers to the exogeneity of the instrumental variables and tests whether the instruments are correlated with the estimated residuals (i.e. it tests if that part of the variation of the endogenous variable captured by the instrumental variables is exogenous) [38].

Finally, identification is a precondition for the application of 2SLS. Specifically, a structural equation ‘is identified only when enough of the system’s predetermined variables are omitted from the equation in question to allow that equation to be distinguished from all the others in the system’ ([39] , p. 430). Both Eqs. 3) and 4) satisfy the necessary condition to be identified (that is, the number of exogenous variables in the system is greater than the number of slope coefficients of each equation). Regression analysis was performed in Stata version 16.1 (Stata Corp LLC, Texas, USA), using the command’ xtivreg2’ to run panel data instrumental variables regressions (and the commands’ fe’ and ‘robust’ to control for country fixed effects and heteroskedasticity, respectively).

## Results

The results of the regression analysis, as summarised in Table 2, indicate the existence of a vicious cycle between the consumption of soft drinks and the prevalence of obesity. The left-hand side of Table 2 collects the regression results based on Eq. 3), in which the dependent variable is *QSD*. All explanatory variables were statistically significant and displayed the expected sign. Specifically, changes in the spread of obesity were associated with changes in soft drink consumption. Holding fixed all other factors affecting *QSD*, a one-unit increase in the age-adjusted prevalence rate of obesity increased the consumption of soft drinks by about 2.39 l per person per year.

**Table 2:** Regression results (2SLS): Obesity, all countries

Dependent variable: Soft drink consumption, ***QSD***				Dependent variable: Prevalence of obesity, ***OBE***			
Independent variables		Coefficient	Std. Error^**1**^	Independent variables		Coefficient	Std. Error^**1**^
Prevalence of obesity	*OBE*	2.390***	0.233	Soft drink consumption	*QSD*	0.069**	0.024
Soft drink price	*PSD*	−2.003***	0.562	Dietary energy supply	*DES*	2.913***	0.667
Income per capita	*GNI*	0.737***	0.157	Urban population	*URB*	0.389***	0.042
Population aged 65 and above	*AGE*	−5.212***	0.496	Population aged 65 and above	*AGE*	1.043***	0.075
Economic globalisation	*GLO*	0.348***	0.089	Economic globalisation	*GLO*	−0.045*	0.017
N. of obs.	1440			N. of obs.	1440		
F-statistic, F(5, 1339)	53.85, Prob. 0.000			F-statistic, F(5, 1339)	304.70, Prob. 0.000		
Underidentification test	188.60, P-val. 0.000			Underidentification test	66.89, P-val. 0.000		
Weak identification test	497.31			Weak identification test	63.32		
Sargan-Hansen J statistic	0.077, P-val. 0.782			Sargan-Hansen J statistic	0.049, P-val. 0.824		

**Notes:** 2SLS: Two-stage least square estimation, with fixed effects

^1^ Heteroskedasticity-robust standard errors. *, ** and *** denote *p* < 0.05, *p* < 0.01 and *p* < 0.001, respectively

Underidentification test (Kleibergen-Paap rk LM statistic), Weak identification test (Cragg-Donald Wald F statistic),

Sargan-Hansen J statistic (overidentification test of all instruments)

Soft drink consumption also responded to changes in both price and income per capita. A one-unit increase in the average price per litre was associated with a decrease in the quantity consumed by around 2 l (per person per year). Conversely, consumption increased by about 0.7 units for each $1000 of additional income per capita, denoting soft drinks as normal goods whose consumption moves in the same direction as income. Moreover, soft drink consumption was inversely related to population aging (i.e. *QSD* decreased by 5.2 l for every 1 % increase in the share of people aged 65 or more in the total population). Finally, globalisation showed a positive relationship with soft drink consumption, which increased by about 0.35 l (per person per year) for every one-unit increase in the degree of economic globalisation as measured by the *KOF* index.

The left-hand side of Table 2 displays the regression results based on Eq. 4), in which the dependent variable is *OBE*. As the consumption of soft drinks rose, so did the prevalence of obesity. Specifically, the age-adjusted rate of obesity increased by 0.07% for every additional litre consumed per capita. The impact of *QSD* on *OBE* was statistically significant. Furthermore, the prevalence of obesity also increased with the amount of energy available for human consumption (*DES*), the share of urban population (*URB*), and the share of elderly people (*AGE*), as expected. In contrast, the impact of the degree of economic globalisation was slightly negative (− 0.04) and also less statistically significant. However, this unexpected sign is consistent with recent research on the effects of globalisation on the spread of obesity, which has documented a complex chain of relationships between obesity and the globalisation process [37].

Results of the statistical tests on the instrumental variables are reported in the bottom lines of Table 2. In both equations, we rejected the null hypothesis that the equations were underidentified, so the instruments chosen were relevant. In addition, we found a first-stage F-statistic larger than the Stock-Yogo critical values in the weak identification test, suggesting that our instruments were not weak. Finally, the *p*-values of the J-statistic for the overidentification test of all instruments for Eqs. 3) and 4) were 0.78 and 0.82, respectively. These results indicate that we cannot reject the null hypothesis that the instrumental variables were valid (i.e. uncorrelated with the error term in the second stage).

Finally, two sensitivity tests were performed. First, we tested the model using the prevalence of overweight (*OWE*). Second, we divided countries into two groups: high-income countries and low- and middle-income countries (that is, low, lower-middle, and upper-middle-income countries), according to the World Bank’s classification of countries by income levels [40].

The results of the first test are collected in Table 3 and confirm the interplay between soft drink consumption and the prevalence of overweight. Moreover, the impact of a one-unit increase in the prevalence of overweight on consumption and, vice-versa, the impact of a one-unit increase in consumption on the prevalence of overweight were, respectively, smaller (1.57 < 2.39) and greater (0.12 > 0.07) than that of obesity, as expected. The statistical tests on the instrumental variables showed that the instruments used were both relevant and exogenous.

**Table 3:** Regression results (2SLS): Overweight, all countries

Dependent variable: Soft drink consumption, ***QSD***				Dependent variable: Prevalence of overweight, ***OWE***			
Independent variables		Coefficient	Std. Error^**1**^	Independent variables		Coefficient	Std. Error^**1**^
Prevalence of overweight	*OWE*	1.576***	0.142	Soft drink consumption	*QSD*	0.119***	0.025
Soft drink price	*PSD*	− 1.744***	0.546	Dietary energy supply	*DES*	4.259***	0.717
Income per capita	*GNI*	0.751***	0.136	Urban population	*URB*	0.590***	0.042
Population aged 65 and above	*AGE*	−4.602***	0.410	Population aged 65 and above	*AGE*	1.199***	0.073
Economic globalisation	*GLO*	0.329***	0.084	Economic globalisation	*GLO*	−0.062***	0.017
N. of obs.	1440			N. of obs.	1440		
F-statistic, F(5, 1339)	61.14, Prob. 0.000			F-statistic, F(5, 1339)	616.46, Prob. 0.000		
Underidentification test	262.73, P-val. 0.000			Underidentification test	66.90, P-val. 0.000		
Weak identification test	13,014.05			Weak identification test	63.31		
Sargan-Hansen J statistic	0.079, P-val. 0.778			Sargan-Hansen J statistic	1.295, P-val. 0.255		

**Notes:** 2SLS: Two-stage least square estimation, with fixed effects

^1^ Heteroskedasticity-robust standard errors. *** denotes *p* < 0.001, respectively

Underidentification test (Kleibergen-Paap rk LM statistic), Weak identification test (Cragg-Donald Wald F statistic),

Sargan-Hansen J statistic (overidentification test of all instruments)

The results of the second test, collected in Tables 4 and 5, show that the two-way relationship between consumption (*QSD*) and obesity (*OBE*) was consistent across both income groups. It is worth noting that in low- and middle-income countries, the impact of a one-unit increase in the prevalence of obesity on the consumption of soft drinks was greater than that observed in high-income countries (1.8 and 1.1 l, respectively). Conversely, a one-unit increase in soft drink consumption increased the prevalence of obesity by about the same amount (around 0.06%) in both groups, as expected. According to all post-estimation tests, the variables chosen were valid instruments and the model was always correctly identified (only in the demand equation for the group of low- and middle-income countries, the overidentification test did not provide the expected result, denoting that one of the instruments was slightly weak).

**Table 4:** Regression results (2SLS): Obesity, high-income countries

Dependent variable: Soft drink consumption, ***QSD***				Dependent variable: Prevalence of obesity, ***OBE***			
Independent variables		Coefficient	Std. Error^**1**^	Independent variables		Coefficient	Std. Error^**1**^
Prevalence of obesity	*OBE*	1.084**	0.406	Soft drink consumption	*QSD*	0.061**	0.022
Soft drink price	*PSD*	−6.764***	1.601	Dietary energy supply	*DES*	4.238***	1.147
Income per capita	*GNI*	0.757***	0.150	Urban population	*URB*	0.553***	0.043
Population aged 65 and above	*AGE*	−4.301***	0.680	Population aged 65 and above	*AGE*	1.001***	0.078
Economic globalisation	*GLO*	0.515**	0.175	Economic globalisation	*GLO*	−0.081**	0.025
N. of obs.	859			N. of obs.	859		
F-statistic, F(5, 790)	21.41, Prob. 0.000			F-statistic, F(5, 790)	233.48, Prob. 0.000		
Underidentification test	78.72, P-val. 0.000			Underidentification test	37.59, P-val. 0.000		
Weak identification test	310.60			Weak identification test	47.02		
Sargan-Hansen J statistic	1.342, P-val. 0.247			Sargan-Hansen J statistic	0.294, P-val. 0.588

**Table 5:** Regression results (2SLS): Obesity, non-high-income countries

Dependent variable: Soft drink consumption, ***QSD***				Dependent variable: Prevalence of obesity, ***OBE***			
Independent variables		Coefficient	Std. Error^**1**^	Independent variables		Coefficient	Std. Error^**1**^
Prevalence of obesity	*OBE*	1.776***	0.270	Soft drink consumption	*QSD*	0.058**	0.029
Soft drink price	*PSD*	−0.628*	0.324	Dietary energy supply	*DES*	2.572***	0.548
Income per capita	*GNI*	2.780***	0.481	Urban population	*URB*	0.389***	0.045
Population aged 65 and above	*AGE*	−1.652	1.185	Population aged 65 and above	*AGE*	0.825***	0.183
Economic globalisation	*GLO*	0.213*	0.086	Economic globalisation	*GLO*	−0.062**	0.019
N. of obs.	578			N. of obs.	578		
F-statistic, F(5, 530)	85.95, Prob. 0.000			F-statistic, F(5, 530)	193.15, Prob. 0.000		
Underidentification test	77.41, P-val. 0.000			Underidentification test	31.36, P-val. 0.000		
Weak identification test	167.47			Weak identification test	45.41		
Sargan-Hansen J statistic	7.303, P-val. 0.007			Sargan-Hansen J statistic	0.164, P-val. 0.685		

**Notes:** Tables 4 and 5. 2SLS: Two-stage least square estimation, with fixed effects

^1^ Heteroskedasticity-robust standard errors. *, ** and *** denote *p* < 0.05, *p* < 0.01 and *p* < 0.001, respectively

Underidentification test (Kleibergen-Paap rk LM statistic), Weak identification test (Cragg-Donald Wald F statistic),

Sargan-Hansen J statistic (overidentification test of all instruments)

### The interplay between soft drinks and obesity

**Fig. 2:**
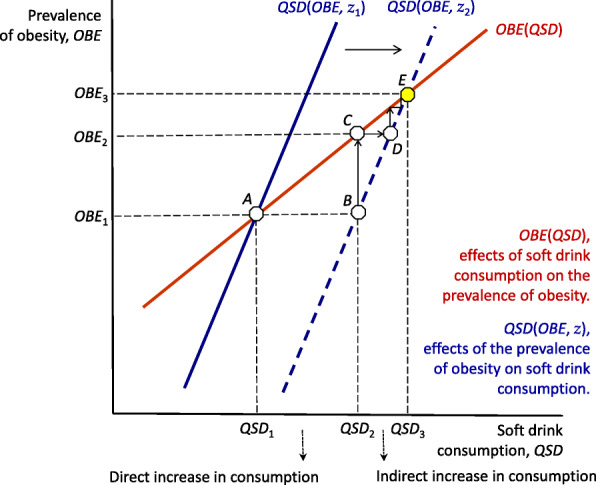
Interplay between soft drink consumption and the prevalence of obesity. Legends: *QSD*, litres/person/year. *OBE*, age-stand. Rate, both sexes, 18+ years, %

The public health implications of the interdependency between the consumption of soft drinks and the prevalence of obesity are summarised in Fig. 2. The horizontal axis measures the per capita consumption of soft drinks (*QSD*), and the vertical axis measures the prevalence of obesity (*OBE*). To simplify, let us consider linear relationships. The impact of soft drink consumption on the spread of obesity (i.e. Eq. 2)) is shown by the solid red curve labelled *OBE*(*QSD*). This function is upward sloping, showing that an increase in the amount of soft drink consumed by the population (i.e. a rightward movement along the x-axis) leads to an increase in the prevalence of obesity.

Conversely, the impact of the prevalence of obesity on soft drink consumption (i.e., Eq. 1)) is shown by the solid blue curve labelled *QSD*(*OBE*, *z*_1_). This function—plotted in the space (*QSD*, *OBE*) for ease of demonstration—is also upward sloping because a higher prevalence of obesity among the population (i.e. an upward movement on the y-axis) leads to a higher demand for soft drinks. In this context, *QSD* and *OBE* are jointly determined. The adoption of unhealthy eating habits (i.e. the consumption of soft drinks) and the spread of their adverse health outcomes (i.e. the prevalence of obesity) influence each other, determining the observed levels of consumption and obesity (*QSD*_1_ and *OBE*_1_, at point A in Fig. 2).

This simplified diagram helps us to better understand the nature of this self-reinforcing process that links the consumption of soft drinks and the prevalence of obesity. Let us consider, for instance, an emerging economy where an increasing per capita income drives a process of nutrition transition from traditional to ultra-processed foods and beverages. In Fig. 2, we denote with *z* a catchall variable that stands for all factors, other than the prevalence of obesity, affecting soft drink consumption (such as the price of soft drinks, consumers’ income, tastes etc.). Thus, a change in *z* (for example, from *z*_1_ to *z*_2_), as a result of the new (and unhealthy) dietary habits, shifts the entire curve *QSD*(*OBE*, *z*) to the right, indicating higher soft drink consumption, at any given prevalence of obesity.

Specifically, *QSD*(*OBE*, *z*) shifts from the original solid blue curve *QSD*(*OBE*, *z*_1_) to the new dotted blue curve *QSD*(*OBE*, *z*_2_). If there were no effects of the prevalence of obesity on the consumption of soft drinks, the increase in *QSD* would simply match the effect of the new dietary pattern. This direct impact is shown by the movement from point A to point B in Fig. 2, which implies an increase in soft drink consumption from *QSD*_1_ to *QSD*_2_. However, this is not the end of the story. Such an increase in *QSD* triggers a feedback loop between consumption and obesity. Given the impact of soft drink consumption on the prevalence of obesity—i.e. given the *OBE*(*QSD*) red curve—a higher level of consumption leads to an increase in the prevalence of obesity.

More specifically, the rise of consumption per capita from *QSD*_1_ to *QSD*_2_ increases the prevalence of obesity among the population from *OBE*_1_ to *OBE*_2_ (shown, in Fig. 2, by the upward movement from point B to point C). In turn, an increase in the spread of obesity implies a higher share of consumers with unhealthy dietary habits. Thus, it promotes a further increase in the consumption of soft drinks (the rightward movement from point C to point D). Again, this leads to a further increase in the prevalence of obesity, and so forth. This process continues, increasing consumption and obesity, until both converge on their new equilibrium levels, *QSD*_3_ and *OBE*_3_ at point E.

In other words, the vicious cycle that links the adoption of unhealthy dietary patterns and the spread of obesity amplifies the impact of any exogenous change in either or both consumption and obesity. In the case shown in Fig. 2, in response to the initial change in consumption from *QSD*_1_ to *QSD*_2_, obesity increases from *OBE*_1_ to *OBE*_3,_ and consumption increases from *QSD*_1_ to *QSD*_3_. This increase in consumption is the sum of two components: the initial direct effect (*QSD*_1_ to *QSD*_2_) plus an indirect effect due to the mutual interaction between *QSD* and *OBE*, measured by the increase from *QSD*_2_ to *QSD*_3_. Similarly, this framework allows analysis of the direct and indirect effects of an exogenous change in the prevalence of obesity for any given level of soft drink consumption due, for instance, to the spread of sedentary lifestyles as a result of the pervasive diffusion of new technologies.

### Impact multipliers

A simultaneous equation model can also be written through reduced-form equations, which express each endogenous variable solely in terms of all the exogenous variables in the system. To this end, using the results collected in Table 2, we rewrite the theoretical regression Eqs. 3) and 4) into their corresponding estimated equations:

5 $$ {QSD}_{it}=2.39{OBE}_{it}-2.00{PSD}_{it}+0.74{GNI}_{it}-5.21{AGE}_{it}+0.35{GLO}_{it}. $$

6 $$ {OBE}_{it}=0.07{QSD}_{it}+2.91{DES}_{it}+0.39{URB}_{it}+1.04{AGE}_{it}-0.04{GLO}_{it}. $$

(where the estimated coefficients are rounded to two decimal places for conciseness). Then, we substitute Eq. 6) into 5) and vice-versa. We thus solve the resulting equations for *QSD* and *OBE* respectively, so as to obtain the following reduced-form equations:

7 $$ {QSD}_{it}=-2.40{PSD}_{it}+0.88{GNI}_{it}-3.26{AGE}_{it}+0.29{GLO}_{it}+8.34{DES}_{it}+1.11{URB}_{it}. $$

8 $$ {OBE}_{it}=-0.17{PSD}_{it}+0.06{GNI}_{it}+0.82{AGE}_{it}-0.02{\mathrm{GLO}}_{it}+3.49{DES}_{it}+0.47{URB}_{it}. $$

The coefficients in the estimated Eqs. 5) and 6) are slope parameters. They measure the response of *QSD* (or *OBE*) to a one-unit increase in the corresponding explanatory variable, holding constant the influence of any other explanatory variables. Instead, the coefficients in Eqs. 7) and 8) are impact multipliers. Each of these coefficients measures the impact on the endogenous variable of a one-unit increase in the value of the corresponding exogenous variable after allowing for the feedback effects from the entire simultaneous system [39].

By comparing slope parameters and impact multipliers, we can assess the role played by the interdependent relationship that links consumption and obesity in promoting the development of an obesogenic food environment. For instance, as documented in the Results section, the direct effect of a one-unit decrease in the average price of soft drinks is an increase in consumption of two litres per person per year. However, the lower price—due, for example, to an aggressive pricing strategy of producers or distributors—leads not only to greater consumption but also to an increase in the spread of obesity, triggering the vicious cycle described in Fig. 2.

The ultimate outcome of a one-unit decrease in *PSD* is twofold: a growth of around 0.17% in the prevalence of obesity (Eq. 8)), and a growth in the consumption of soft drinks of around 2.4 l per person (Eq. 7)), that is, a final quantity consumed that is greater than the initial increase caused directly by the price reduction. Thus, the difference between the impact multiplier of *PSD* in Eq. 7) and the slope parameter of *PSD* in Eq. 5)—that is, 2.4–2.0 = 0.4—measures the indirect impact of a price change on consumption. That is to say, the feedback loop between soft drinks and obesity adds 0.4 l of extra consumption for every one-unit decrease in the average price of soft drinks.

## Discussion

Existing studies have examined the association between soft drinks and obesity using cross-country datasets [20–23]. Our results are in line with those observed in these previous studies. In reviewing the literature, however, we found very little about a feedback process in which the consumption of soft drinks and the prevalence of obesity interact and reinforce each other. This work was designed to investigate the hypothesis that the consumption of calorie-dense beverages and the prevalence of obesity are jointly determined. We found that soft drinks and obesity are linked in a vicious cycle. This result indicates that the development of obesogenic food environments should be considered as a self-reinforcing process.

Although the purpose of this study was to document whether and to what extent dietary habits and weight outcomes influence each other, we attempt to introduce some hypotheses about the potential mechanisms behind this vicious cycle. In the food industry, firms engage in both price and non-price competition. For those firms that produce ultra-processed foods and beverages, product development is a crucial strategy of non-price competition [41]. In order to increase product differentiation, this strategy is often based on the improvement of palatability (i.e. the positive hedonic evaluation of foods’ characteristics) and, more generally, on the improvement of the product’s sensory properties (such as sight, smell, taste, and texture), by adding sugars, fats, salt, and various additives during processing [42].

In other words, this kind of competition tends to increase the number and variety of highly palatable and unhealthy calorie-dense foods and beverages [43]. Usually, such products are not only nutritionally unbalanced but also habit-forming (or even addictive) [44]. As a result, the interaction between firms’ strategies and consumers’ preferences triggers a vicious cycle. The wide availability of convenient and affordable unhealthy products promotes the adoption of unhealthy dietary patterns. In turn, the regular consumption of these habit-forming foods and beverages shapes consumers’ tastes and purchasing behaviours, thereby increasing the demand for unhealthy products, and so on.

In the case of soft drinks, these products are usually the largest source of calories and added sugar in the diet of millions of people, especially in high- and middle-income countries [45, 46]. There is strong evidence that the regular consumption of soft drinks increases the risk of overweight and obesity among children, adolescents, and adults [16]. Research has also shown the potentially addictive properties of the high amount of sugar contained in the large majority of soft drinks [47, 48]. Furthermore, recent studies in behavioural sciences suggest the existence of a vicious cycle between obesity and cognitive dysfunction [49, 50]. This vicious cycle is triggered by adopting a Western diet, mainly based on energy-dense foods and beverages (such as SSBs). It results in the following sequence: overconsumption of unhealthy ultra-processed products, positive energy balance, hippocampal dysfunction, impaired inhibitory cognitive control of responding to environmental food cues, further overconsumption of unhealthy products, and so forth. We also know [51] that obese patients face barriers to change their eating habits towards healthy diets (such as lack of willpower, time constraints, taste preferences, and the pervasive availability of unhealthy foods and beverages). From the demand side, this implies that the increase in the prevalence of obesity, caused by the regular consumption of soft drinks, creates the ideal conditions for further market expansions, promoting the consumption of soft drinks and other calorie-dense products in the everyday diet. From the supply side, this vicious cycle is further reinforced by the price and non-price strategies of the beverage industry that increase affordability and availability of a wide range of soft drinks, enhancing the development of an obesogenic food environment.

From a public health perspective, our results further support the use of fiscal policies, such as taxes on sugar-sweetened beverages (SSBs), to curb the consumption of these calorie-dense products [52]. Specifically, the identification of a feedback loop involving consumption and obesity may imply that the long-run effects on the prevalence of obesity of an excise tax (that raises the market price of SSBs) are probably currently underestimated.

Some recent papers have tried to model the potential impacts of ‘sugar taxes’ on health outcomes. Albeit based on the single causal relationship between consumption and obesity (and limited to simulation studies), these papers have consistently shown that taxes can lead to reductions in incident rates of obesity [53]. However, taxes on SSBs are designed to decrease consumption but also to raise public awareness, incentive product reformulation, and generate government revenue (to be used, for instance, to implement nutrition education programs). Conversely, considering the two-way interplay between consumption and obesity, one can see that all these potential outcomes shift either or both the *QSD*(*OBE*) and *OBE*(*QSD*) relationships. For instance, despite the small direct impact of *QSD* on *OBE* (i.e. one-litre change in per capita consumption of soft drinks will change obesity prevalence by 0.07%), the total (direct and indirect) effect on the prevalence of obesity of a one-unit increase in the average price of soft drinks in around 2.5 times larger than that indicated by the slope parameter of the single demand equation model. As a result, introducing a tax on SSBs can trigger a virtuous cycle that amplifies the initial decrease in consumption and obesity.

### Limitations

Several limitations need to be noted regarding the results of the regression analysis. First, the consumption of soft drinks and the prevalence of obesity are affected by many factors. Our empirical model is based on a very small set of variables. This limitation is mainly due to a lack of data. For many countries, especially low-income countries, no reliable data exist, or the available data span too short a period of time to be used in a balanced panel data set. This leads to a second important weakness. Both soft drink consumption and the prevalence of obesity are growing phenomena in low- and lower-middle-income countries [54, 55]. Our dataset, however, included only three low-income countries and 23 lower-middle-income countries (conversely, it included 72 upper-middle and high-income countries, as shown in Table 1A in the Supporting Information File S1). Third, for the same reasons (i.e. data availability), we were not able to account for the effects of income inequalities on dietary patterns and health outcomes even within upper-middle and high-income countries.

Furthermore, data on the dependent variables (i.e. the prevalence of overweight/obesity, and the consumption of soft drinks) are not fully comparable. The variables *OBE* and *OWE* are estimated from population-based data [56], whereas *QSD* comes from sales data that are only a proxy of the effective consumption, due to the increasing phenomenon of food waste [57]. In addition, *QSD* measures sales per capita of a wide range of beverages that contain very different quantities of free sugars (e.g. some regular sodas contain less than 10 g of sugar per eight oz. serving, and others more than 45 g) [58]. Moreover, in recent years, taxes on sugar-sweetened beverages have been implemented in more than 35 countries worldwide [52, 53], and, more generally, many countries have implemented obesity prevention programs [7]. These national food policies are a potential confounding factor in the interplay between soft drinks and obesity, because they may lead to exogenous shifts in both *QSD*(*OBE*) and *OBE*(*QSD*) relationships.

Another potential weakness comes from the use of a country fixed effects model, which did not allow us to control for variables that change over time. However, random effects models are based on more stringent assumptions and are more challenging to estimate and interpret. Therefore, as a first attempt to study soft drink consumption and the prevalence of obesity within a simultaneous equation model, we preferred to be more conservative and control only for country-specific fixed effects. Finally, we found associations that did not necessarily indicate the existence of cause-and-effect relationships, and the use of country-level data offered the potential for ecological fallacies. However, this is the first study to document a two-way relationship between soft drinks and obesity. Further research should be undertaken to apply a simultaneous equation model to individual data, using a broader range of control variables and different types of ultra-processed foods and beverages.

## Conclusions

Obesity is a complex and multifactorial disease. The onset of obesity, however, is largely preventable. Relatively simple changes in dietary habits and physical activity effectively help to maintain a healthy weight, reducing the risk of developing one or more obesity-related diseases [59]. Nevertheless, dietary habits are strongly influenced by the food environment within which people make their everyday consumption choices. Making healthy choices is challenging within obesogenic food environments, where affordable, convenient, nutritionally unbalanced, hyper-palatable, and habit-forming ultra-process foods and beverages are ubiquitous and aggressively marketed. However, the development of an obesogenic environment is itself both a cause and an effect of the spread of unhealthy habits and behaviours. This study has identified a vicious cycle between unhealthy habits (i.e. consuming soft drinks) and health outcomes (i.e. the prevalence of obesity). We documented that the consumption of soft drinks and the prevalence of obesity influence each other. This interplay amplifies the impact of any exogenous changes in the determinants of consumption and obesity. A change in the price of soft drinks, for instance, affects not only the quantity consumed but also the spread of obesity, leading to an overall change in consumption greater than the one due to the direct effect of the initial price change. These feedback effects should be considered and exploited in planning effective strategies to tackle the burden of obesity and the NCDs epidemic.

## Supplementary Information

**Additional file 1.** List of variables. Table 1A. List of countries included in the study by income group. Table 2A. Dataset.

## Data Availability

We used secondary data. All data generated or analysed during this study are included in this published article and in the Supporting Information File S1. The full dataset is also publicly available at Mendeley Data (10.17632/hkm25rbpsc.2).
